# Combined Transcriptomics and Metabolomics Analysis Reveals the Molecular Mechanism of Salt Tolerance of Huayouza 62, an Elite Cultivar in Rapeseed (*Brassica napus* L.)

**DOI:** 10.3390/ijms23031279

**Published:** 2022-01-24

**Authors:** Heping Wan, Jiali Qian, Hao Zhang, Hongchen Lu, Ouqi Li, Rihui Li, Yi Yu, Jing Wen, Lun Zhao, Bin Yi, Tingdong Fu, Jinxiong Shen

**Affiliations:** 1 National Key Laboratory of Crop Genetic Improvement, National Engineering Research Center of Rapeseed, College of Plant Science and Technology, Huazhong Agricultural University, Wuhan 430070, China; wanheping@jhun.edu.cn (H.W.); qianjiali@vazyme.com (J.Q.); haozhang1108@163.com (H.Z.); zjluhongchen@163.com (H.L.); oql1119@163.com (O.L.); lirh20212021@163.com (R.L.); wenjing@mail.hzau.edu.cn (J.W.); zhaolun@mail.hzau.edu.cn (L.Z.); yibin@mail.hzau.edu.cn (B.Y.); futing@mail.hzau.edu.cn (T.F.); 2 Hubei Engineering Research Center for Protection and Utilization of Special Biological Resources in the Hanjiang River Basin, School of Life Science, Jianghan University, Wuhan 430056, China; yuyixxx@yeah.net

**Keywords:** *Brassica napus*, transcriptomics, metabolomics, salt stress, *BnLTP3*

## Abstract

Soil salinity is one of the most significant abiotic stresses affecting crop yield around the world. To explore the molecular mechanism of salt tolerance in rapeseed (*Brassica napus* L.), the transcriptome analysis and metabolomics analysis were used to dissect the differentially expressed genes and metabolites in two rapeseed varieties with significant differences in salt tolerance; one is an elite rapeseed cultivar, Huayouza 62. A total of 103 key differentially expressed metabolites (DEMs) and 53 key differentials expressed genes (DEGs) that might be related to salt stress were identified through metabolomics and transcriptomics analysis. GO and KEGG analysis revealed that the DEGs were mainly involved in ion transport, reactive oxygen scavenging, osmotic regulation substance synthesis, and macromolecular protein synthesis. The DEMs were involved in TCA cycle, proline metabolism, inositol metabolism, carbohydrate metabolic processes, and oxidation-reduction processes. In addition, overexpression of *BnLTP3*, which was one of the key DEGs, could increase tolerance to salt stress in *Arabidopsis* plants. This study reveals that the regulation mechanism of salt tolerance in rapeseed at the transcriptome and metabolism level and provides abundant data for further in-depth identification of essential salt tolerance genes.

## 1. Introduction

Soil salinity is one of the most significant abiotic stresses affecting seed germination, crop growth, and productivity [1]. According to a rough estimate, approximately 280 million hectares of agricultural land are affected by salt stress, and this problem continues to worsen [2]. It is predicted that over 50% of all agricultural land will be threatened by 2050 [3]. Thus, understanding the crop salt tolerance mechanisms and breeding high salt-tolerant crops has been of great significance to the sustainable development of world agriculture.

Soil salinity can result in early-occurring osmotic stress and the accumulation of toxic ions in plants [4]. In order to reduce the salt damage, plants have four main mechanisms to cope with salt stress. The first category is osmotic regulation, and there are two osmotic adjustment methods in plants. One is to absorb and accumulate inorganic ions such as Na^+^, K^+^, Cl^−^, Ca^2+^, and Mg^2+^ in the cell, and the other is to accumulate small molecules such as proline, betaine, polyols, sugars, and other osmotic substances in cells [5]. The second category is the ion balance regulation, which regulates the ion balance through ion transporters, and channels, and maintains the stable state of ions in cells and tissues [6]. The third category is that plants produce and accumulate specific macromolecular proteins that are induced and expressed by high salt, such as common osmolarity (OSM) and aquaporins (AQP) and late embryogenesis-abundant protein (LEA) [5]. The fourth category is active oxygen scavenging methods. The scavenging of excessive ROS under high salinity may be attributed to non-enzymatic antioxidant metabolites, including ascorbate, glutathione, and tocopherols, and enzymatic agents, such as catalases (CAT), superoxide dismutase (SOD), ascorbate peroxidase (APX), and glutathione reductase (GR) [7]. A large number of salt-responsive genes have been identified, such as *SNC1*, which encoded a high-affinity K^+^ transporter (HKT)-type sodium transporter [6], and salt-response genes include transcription factors (TFs), such as ERF and WRKY, and signal-related protein kinase [8]. By combining the interaction mode among the mentioned genes above, several pathways that improved the salt-stress signal have been revealed, such as the salt overly sensitive (SOS) pathway [7,8,9,10], the calcium-dependent protein kinase (CDPK) pathway [11], and the mitogen-activated protein kinase (MAPK) pathway [12].

Most crop species, such as rice, maize, and barley, are not salt-tolerant and are adversely affected by high salt stress [1]. Rapeseed (*Brassica napus* L.), one of the most important oil crops all over the world, is regarded as a middle salt-tolerant crop [13]. A series of research advances have been made in researching the genetic mechanism of salt tolerance in rapeseed, and many QTLs and genes related to salt tolerance in rapeseed have been identified. For example, the expression levels of *BnBDC1*, *BnLEA4*, *BnMPK3*, and *BnNAC2* were up-regulated under salt stress [14]. Over-expression of *BnaAOX1b* significantly improved seed germination under salt stress [15]. Although a number of genes related to salt tolerance in rapeseed have been identified, the function and regulatory network of the genes are still unclear. Therefore, a more comprehensive and in-depth study on the molecular mechanism of rapeseed salt tolerance is needed.

With the rapid development of high-throughput sequencing technology, transcriptomics analysis (RNA-seq) has become been successfully employed to explore the molecular mechanism of salt tolerance in different crops, such as rice [16,17] and wheat [18]. To date, limited transcriptome information in response to salt stress in rapeseed has been reported. For example, Long [19] performed RNA-seq to identify 163 DEGs at 0, 3, 12, and 24 h after NaCl treatments on rapeseed roots at the germination stage, such as the glycine-rich protein, ERD family proteins, glycosyltransferase family, and ubiquitin-protein ligase. Yong [20] used RNA-seq to perform comparative transcriptome analysis of leaves and roots in response to salt stress in rapeseed, and a total of 582 transcription factors and 438 transporter genes were differentially regulated in both organs in response to salt stress. The successful application of transcriptomics analysis in the gene mining of salt tolerance traits in rapeseed has shown excellent efficiency in gene detection.

Metabolomics is a quantitative analysis of all metabolites in living organisms and finds the relative relationship between metabolites and physiological and pathological changes [21,22]. Metabolomic analysis can detect small molecular substances and exogenous substances, where small molecular substances include endogenous substances in tissues or organs, and the relative molecular mass is <1000 [23]. Metabolites are the final products of cell-regulation processes, and their levels can be regarded as the final response of biological systems to genetic or environmental changes [24]. In recent years, metabolomics research methods have been successfully applied to analyze the salt tolerance mechanism of different crops, such as maize [25,26], barley [27], and peanut [28], and a large number of metabolites have been identified, such as alanine, glutamate, asparagine, glycine-betaine, sucrose, malic acid, trans-aconitic acid, and glucose. For example, when plants respond to salt stress, the osmotic regulators like proline content and soluble sugars such as glucose, fructose, and sucrose in plants increases significantly, and the plant accumulates more energy metabolites such as phenylalanine, aspartic acid, citric acid, and citramalic acid under salt stress [25,26,27,28]. However, the metabolites in response to salt stress in rapeseed are still uncle ar.

The Huayouza62 (H62) cultivar, which is considered a salt-tolerant rapeseed variety, has been widely planted in the main production areas of rapeseed in the Yangtze River basin of China, and in saline-alkali land in the Gansu, Xinjiang, and Inner Mongolia provinces of China. In addition, H62 has been extended to countries such as Outer Mongolia and Russia. However, the molecular mechanism of salt tolerance in H62 rapeseed is still unclear. Zhongshuang 11 (ZS11) is a conventional rapeseed variety, which is sensitive to salt stress [29]. In this study, the salt-tolerant variety (H62) and salt-sensitive variety (ZS11) were selected, and RNA-seq and metabolomics analysis methods were employed to analyze key salt stress response genes and metabolites in rapeseed under salt stress at the germination stage. This study provides plenty of valuable information about how rapeseed responds to salt stress at the transcriptome and metabolism level.

## 2. Results

### 2.1. Effects of Different NaCl Concentrations on the Germination of Rapeseed Seeds

**Figure 1:**
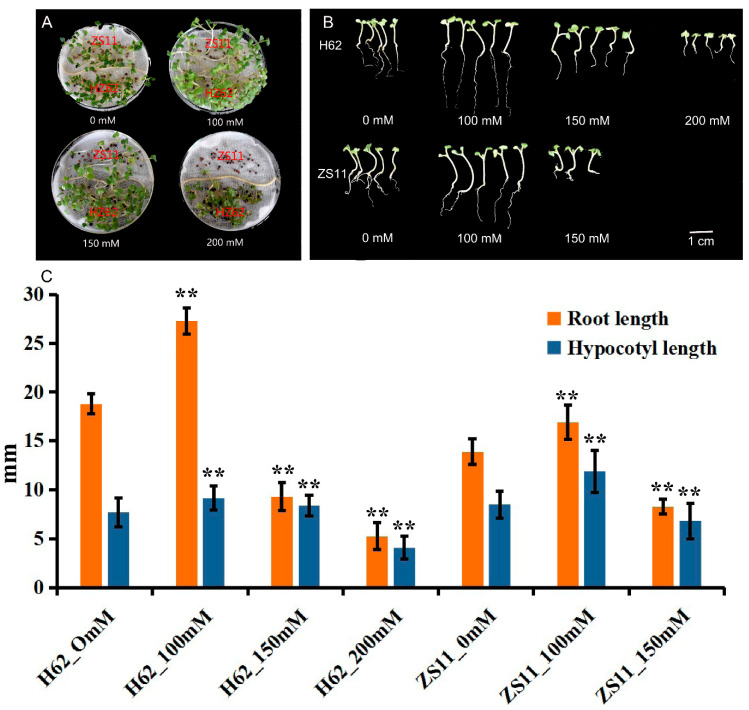
The germination and seedlings grow of two rapeseed varieties (H62 and ZS11) under different NaCl concentrations (0, 100, 150 and 200 mM) on the eighth day. (**A**) Comparison of germination of H62 and ZS11 under different salt concentrations; (**B**) Comparison of early seedlings of H62 and ZS11 under different salt concentrations; (**C**) Hypocotyl length and root length of early seedlings of H62 and ZS11 at different salt concentrations. ** indicates that the root length and hypocotyl length of each variety (H62 and ZS11) under salt stress were significantly different from those of the control condition at *p* < 0.01 (Student’s *t* test).

The germination of rapeseed was affected under different NaCl concentrations from 0 to 200 mM (0, 100, 150, and 200 mM) (Figure 1A). The majority of seeds, both ZS11 and H62, normally germinated under low NaCl (100 and 150 mM)-concentration treatments. When the concentration reached 200 mM, the seeds of ZS11 did not germinate, while the germination rate of H62 reached 90%. The root length (RL) and hypocotyl length (HL) under a low NaCl concentration (100 mM) were higher than the RL and HL of the control sample (Figure 1B). When NaCl concentration reached 150 mM and 200 mM, the RL and HL of seedlings were significantly lower than the RL and HL of the control (Figure 1C). All the above results suggested that there are significant differences in salt tolerance between the H62 and ZS11. Additionally, H62 could be regarded as a salt-tolerant variety, and ZS11 could be regarded as a salt-sensitive variety.

### 2.2. DEMs Description of Two Rapeseed Varieties under Salt Stress

PCA analysis and PLS-DA analysis were performed to analyze the mass spectrometry data of the treatment group. The results showed that the seven biological repeats data points in the treatment group could basically be collected together and the data points of the samples of the two varieties of the treatment groups could be clearly distinguished in space, indicating that the metabolites of each group of samples were different in terms of species, quantity, and concentration (Figure S1).

**Figure 2:**
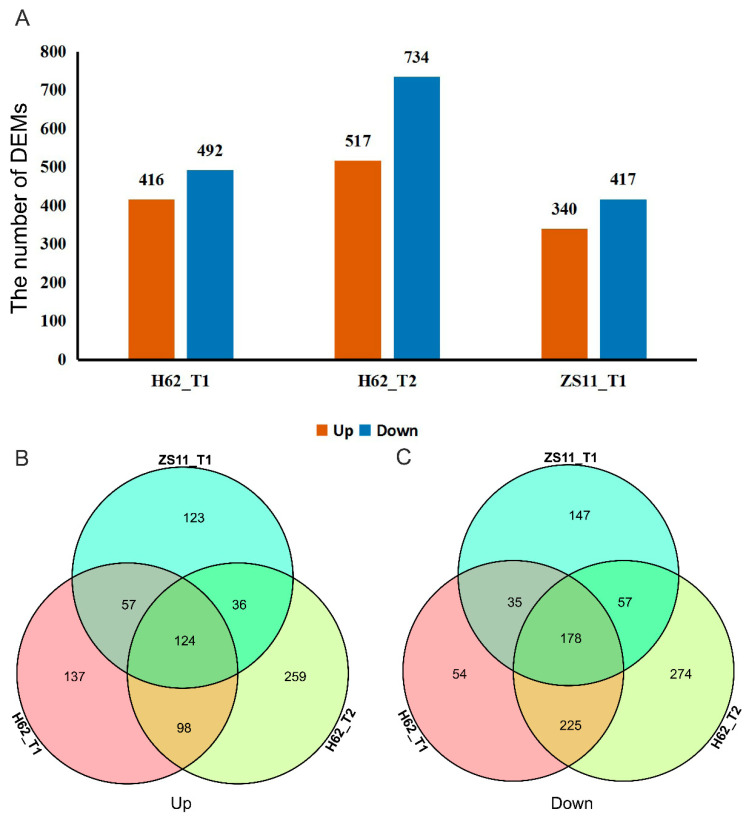
The differentially expressed metabolites (DEMs) among the groups for H62 and ZS11 under salt stress (T) and normal (C) conditions at different NaCl concentrations (T1, 150 mM, T2, 200 mM). (**A**) The number of DEMs in three groups. (**B**) Venn diagram of up-regulated DEMs in three groups. (**C**) Venn diagram of down-regulated DEMs in three groups.

Conditions for screening differentially expressed metabolites (DEMs): (1) VIP ≥ 1; (2) Fold-change ≥ 1.2 or Fold-change ≤ 0.833; (3) q-value < 0.05. 912, 1251, and 757 differentially expressed metabolites (DEMs) were detected in three groups (H62_T1 vs. H62_CK, H62_T2 vs. H62_CK and ZS11_T1 vs. ZS11_CK), respectively, and most DEMs were uniquely associated with a specific salt treatment (Figure 2A). For example, compared with H62_CK, 196 and 295 DEMs were only specifically up-regulated in H62_T1 and H62_T2, respectively, and 222 DEMs were up-regulated in both H62_T1 and H62_T2. Considering the common DEMs in H62_T1, H62_T2, and ZS11_T1, 124 and 178 DEMs were up-regulated and down-regulated in all the salt treatment including H62_T1, H62_T2 and ZS11_T1, respectively, and these DEMs were considered as the salt-related metabolites in rapeseed (Figure 2B,C). The enrichment analysis of the KEGG pathway showed that most of the DEMs were mainly enriched in metabolic pathways, and the biosynthesis of secondary metabolites (Table S2).

**Figure 3:**
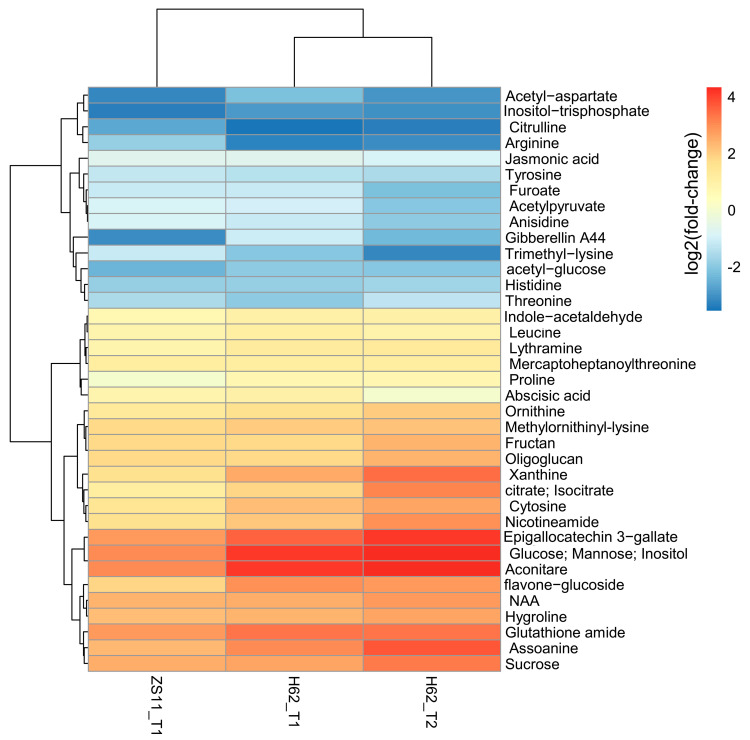
The heat map of the part of DEMs related to salt stress base on the log_2_Foldchange among the groups for H62 and ZS11 under salt stress (T) and normal (C) conditions at different NaCl concentrations (T1, 150 mM, T2, 200 mM).

Combining the pathway function enrichment analysis results, the result of DEMs annotation, and the fold-change levels of the two varieties under different salt concentrations, 103 DEMs were screened out (Table S3), and some DEMs are listed in Figure 3. These DEMs can be divided into several categories. The first group is amino acid, including Proline, Arginine, Ornithine, Leucine, Threonine, Aspartate, and Hygroline. Except for Proline, Ornithine, and Hygroline, which were highly induced by salt stress, the content of other amino acids was significantly down-regulated, suggesting that salt stress could inhibit the normal metabolic process of amino acids in rapeseed. The second group was carbohydrate, including Glucose, Fructan, Inositol, Sucrose, Mannose, and Oligoglucan; most of these were up-regulated by salt stress in two rapeseed. The third group was plant hormone, including ABA, NAA, GA, and JA. NAA was up-regulated and ABA, and GA and JA were down-regulated in H62_T1, H62_T2, and ZS11_T1. Several important metabolic intermediates in the TCA cycle were all up-regulated, such as Citrate, Aconitare, and Isocitrate, indicating that rapeseed cloud enhances its energy metabolism pathway under salt stress.

### 2.3. Sequencing Output and Assembly

Under treatment and control conditions, 15 sequencing libraries were constructed from *Brassica napus* H62 and ZS11 samples at three concentrations (0, 150, 200 mM NaCl) (Table 1). 54.03 Mb to 57.14 Mb of total raw reads were obtained from each of the 15 libraries. The software SOAPnuke (v1.5.2) (https://github.com/BGI-flexlab/SOAPnuke, accessed on 25 March 2019) was used to filter reads, followed as: (1) Remove reads with adaptors; (2) Remove reads in which unknown bases (N) are more than 5%; (3) Remove low-quality reads (the percentage of base whose quality is lesser than 15 is greater than 20% in a read). After filtering, it was confirmed that >80% of the sequences were clean reads. The proportion of filtered reads (CRR) exceeded 75%, and most of them exceeded 80%. The proportion of clean reads (TMR) of the reference genome on the comparison exceeded 70%, and the total number of genes (TGN) exceeded 70,000, so the sequence data were sufficient for gene expression analysis.

### 2.4. General Gene-Expression Description under Salt Stress

**Figure 4:**
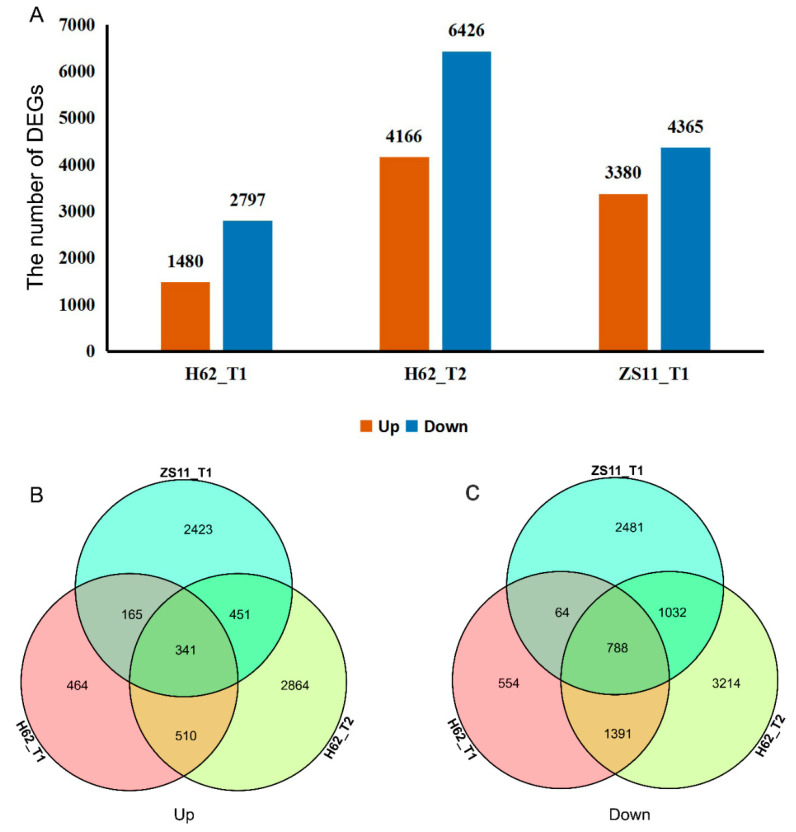
The differentially expressed genes (DEGs) among the groups for H62 and ZS11 under salt stress (T) and normal (C) conditions at different NaCl concentrations (T1, 150 mM, T2, 200 mM). (**A**) The number of DEGs in three groups. (**B**) Venn diagram of up-regulated DEGs in three groups. (**C**) Venn diagram of down-regulated DEGs in three groups.

Adjust *p*-value ≤ 0.05 and a |log_2_FoldChange| ≥ 1 were set as the thresholds to determine the significance of the gene-expression difference between samples. Compared with H62_CK, there were 4277 (Up 1480 and Down 2797) and 10,592 (Up 4166 and Down 6426) DEGs at the treatments of H62_T1 and H62_T2, displaying a rising tendency in DEGs numbers. Compared with ZS11_CK, there were 7745 (Up 3380 and Down 4365) DEGs at the treatments of ZS11_T1 (Figure 4A). Down-regulated genes had greater numbers than those of the up-regulated ones, H62 and ZS11, under different NaCl concentrations. Comparing the number of DEGs of H62 and ZS11 under two salt-concentration conditions, the number of DEGs of H62_T2 and ZS11_T1 were significantly higher than that of H62_T2, providing evidence of gene transcription level for the difference in salt tolerance between H62 and ZS11.

Most DEGs were uniquely associated with a specific salt treatment. For example, compared with H62_CK, 464 and 2864 DEGs were only specifically up-regulated in H62_T1 and H62_T2, respectively, and 851 DEGs were up-regulated in both H62_T1 and H62_T2 (Figure 4B). Considering the common DEGs in H62_T1, H62_T2, and ZS11_T1, 341 and 788 DEGs were up-regulated and down-regulated in all the salt treatments including H62_T1, H62_T2, and ZS11_T1 (Figure 4A–C), and these DEGs were considered as the possible salt-related genes.

### 2.5. GO and KEGG Analysis of DEGs Two Rapeseed Varieties under Different NaCl Condition

**Figure 5:**
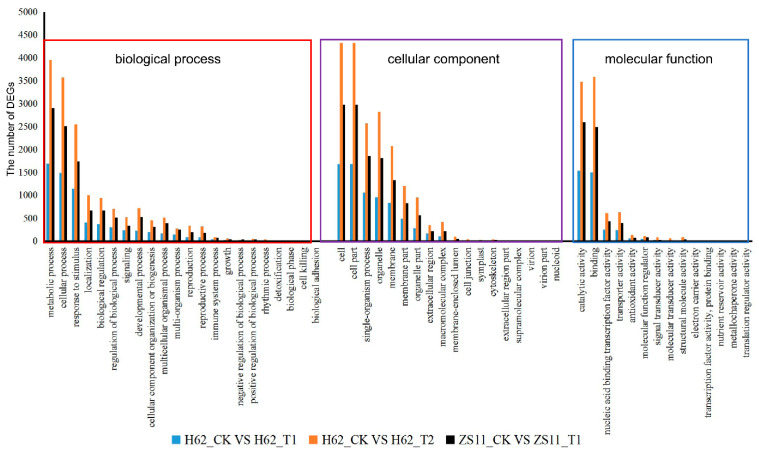
Gene Ontology (GO) analysis of common DEGs of two rapeseed varieties under different NaCl conditions. The DEGs were assigned into three groups, including biological process, cellular components, and molecular function. The *x*-axis represents the most abundant categories of each group, and the *y*-axis represents the percentages of the total genes in each category. Only partial results are shown.

Gene Ontology (GO) assignments were used to classify the functions of the DEGs responding to salt stress. Three non-mutually exclusive GO categories, biological process (BP), cellular component (CC), and molecular function (MF), were well represented. The most represented GO terms are presented in Figure 5. In the BP category, the most abundant GO terms were “metabolic process”, “cellular process”, and “response to stimulus”. In the CC category, “cell” was the most abundant, followed by “cell part”, “organelle”, and “membrane”. In the MF category, “catalytic activity” was the most abundant category, followed by “binding”, “nucleic acid binding transcription factor activity”, “transporter activity”, and “antioxidant activity”. Notably, some genes were assigned to more than one category.

**Figure 6:**
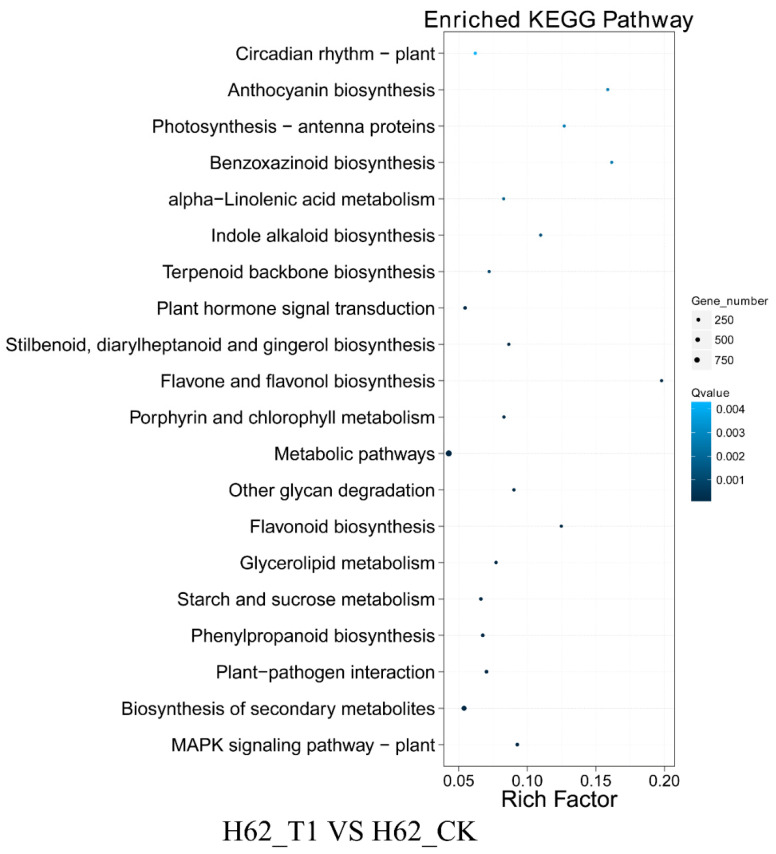
Kyoto Encyclopedia of Genes and Genomes (KEGG) pathway analysis of the DEGs in H62_T1 vs. H62_CK. Only partial results are shown in Figure 6.

According to the results of KEGG, a total of 3261, 7897, and 5856 DEGs could be aligned to the KEGG pathways in H62_T1 vs. H62_CK, H62_T2 vs. H62_CK, and ZS11_T1 vs. ZS11_CK, respectively (Table S4). The pathways with more mapped genes were the metabolic pathways, biosynthesis of secondary metabolites, and starch and sucrose biosynthesis (Figure 6 and Figure S2). In addition, the pathways with more mapped genes were the alanine, aspartate and glutamate metabolism, inositol phosphate metabolism, and fructose, MAPK signaling pathway-plant, and mannose metabolism (Figure 6 and Figure S2).

### 2.6. Identification of Genes Responding to Salt Stress

**Figure 7:**
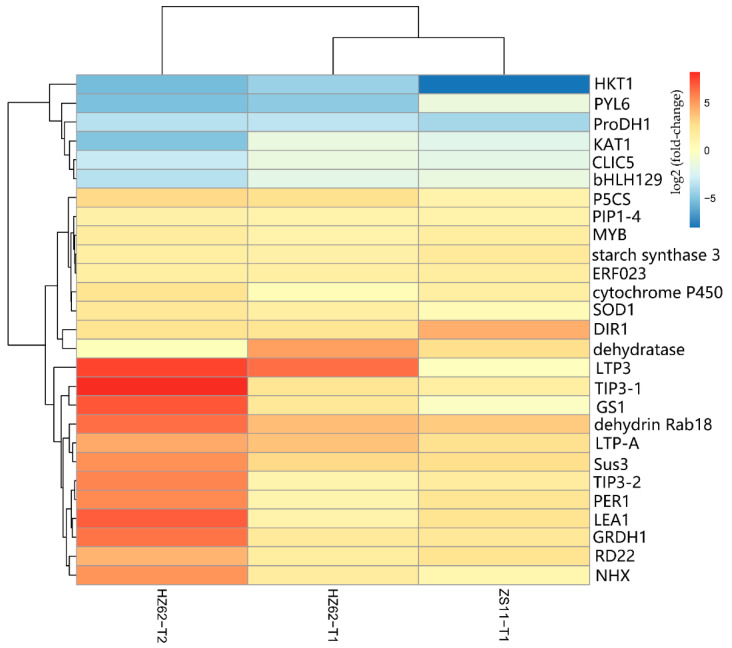
The heat map of part of the candidate genes related to salt stress base on the log_2_Foldchange among the groups for H62 and ZS11 under salt stress (T) and normal (C) conditions at different NaCl concentrations (T1, 150 mM, T2, 200 mM).

A total of 341 genes were up-regulated in H62_T1, H62_T2, and ZS11_T1, and a total of 788 genes were down-regulated in H62_T1, H62_T2, and ZS11_T1 (Figure 4). These genes can be considered as responses to salt stress in rapeseed. Combining the pathway function enrichment analysis results, the result of DEGs function annotation, and the expression levels of the two varieties under different salt concentrations, 53 candidate genes were screened out (Table S5), and some candidate genes are listed in Figure 7. These genes can be divided into several categories, such as ion-channel protein gene (*HKT1*, *CLIC5*, *NHX2*, *NHX4*, *KAT1*), osmotic regulation-related genes (*P5CS*, *P5C reductase*, *ProDH1*, *Sus3, MIPS*), transcription factor (*ERF023*, *ERF018*, *ABI3*, *ETC3, WRKY29*, *MYB39*, *bHLH122*), macromolecular protein genes (*RD22*, *DIR1*, *PIP1-4*, *TIP3-2*, *LEA1*, *LEA76*, *Rab18*), reactive oxygen-scavenging enzyme gene (*SOD1*, *POD34*, *PER1*), and functional enzyme (*GS1*, *SAG12*, *ADH*) (Figure 7, Table S5).

### 2.7. Confirmation of DEG Profiles by qPCR Analysis

**Figure 8:**
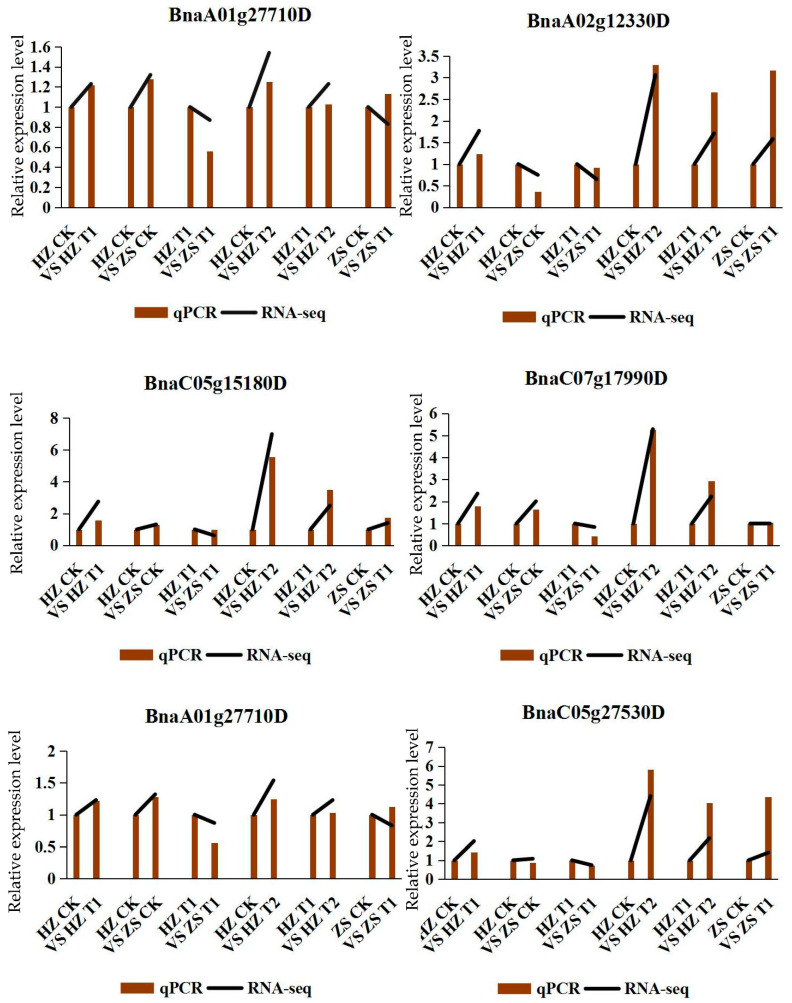
The relative expression of qRT-PCR and RNA-seq of six DEGs.

In order to validate the reliability of our RNA-seq data, we measured mRNA abundance using qRT-PCR for 6 DEGs. All the six tested genes by qRT-PCR were significantly changed between H62 and ZS11 rapeseed under the different NaCl treatments, which was similar to the result observed through RNA-seq analysis (Figure 8). Although the change folds were not exactly the same as those revealed by the transcriptome-profiling data, all the validated genes showed similar expression patterns considering the DEGs data.

### 2.8. Over-Expression of BnLTP3 Increased Salt Tolerance in Arabidopsis thaliana

**Figure 9:**
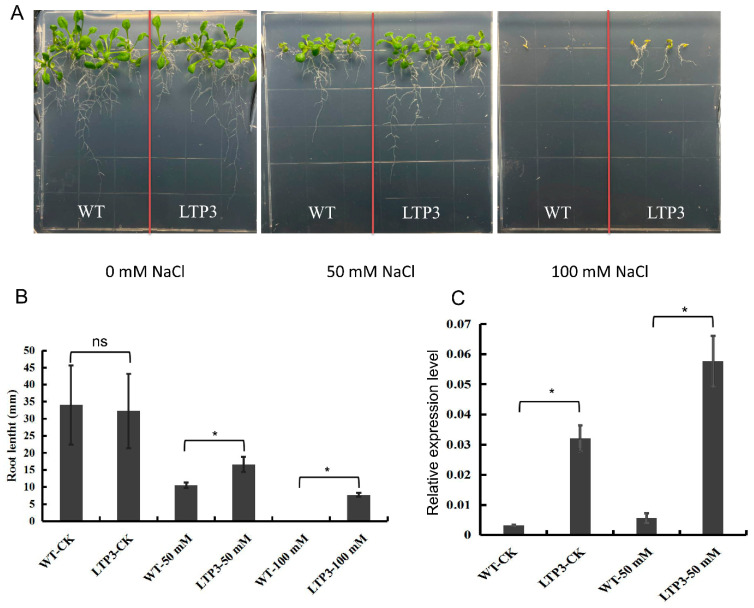
Over-expression of *BnLTP3* in *Arabidopsis thaliana.* (**A**) Phenotypes of WT (wild-type) and *LTP3*-overexpressing plants grown on MS and MS + NaCl (50, 100 mM) media. (**B**) Root lengths of WT and *LTP3* overexpressing plants on MS + NaCl medium (50, 100 mM). Values are means ± SD (*n* = 3); * *p* < 0.05 (Student’s *t* test); ns not significant. (**C**) *LTP3* gene expression level of WT and *LTP3*-overexpressing plants grown on MS and MS + NaCl (50, 100 mM) media.

A number of candidate genes homologous to *LTPs* were also identified. The expression levels of lots of *LTPs* were up-regulated significantly as the salt concentration increased in salt-tolerant H62, but was not significantly up-regulated in ZS11 (Figure S3). In order to determine that the *BnLTP3* is related to salt tolerance, it was transferred to *Arabidopsis thaliana*. The result showed no significant difference between the root length of the wild-type and over-expression lines under 0 mM NaCl (Figure 9 and Figure S4). While, the root lengths of the over-expression lines were significantly higher than that of the wild-type under salt stress conditions (50 mM and 100 mM), indicating that *BnLTP3* has the function of improving salt tolerance (Figure 9).

## 3. Discussion

With the rapid development of modern molecular biology, the research on the mechanism of plant salt tolerance has reached the level of transcriptome, proteome, metabolome, and ionome [30]. The “omics” research provides a powerful method for identifying salt-tolerant genes and the mining of marker metabolites [31]. Unique genetic resources are the genetic basis for omics research. In our study, two distinctive rapeseed varieties (Huayouza 62 and Zhongshuang 11) were selected for transcriptome and metabolome analysis. Huayouza 62 is an elite hybrid rapeseed variety, successfully promoted and planted in China’s saline-alkali land due to its relatively strong salt tolerance. Zhongshuang 11 is an elite conventional rapeseed variety; however, it is very sensitive to salt stress [29]. Our results showed a significant difference between H62 and ZS11 in salt tolerance at the early-seedling stage (Figure 1). Therefore, these two rapeseed varieties are ideal resources for identifying salt-tolerant genes and studying salt-tolerant mechanisms in rapeseed.

Plant accumulate inorganic ions such as Na^+^, K^+^, and Ca^2+^ in the cell, and accumulate small molecules such as proline, betaine, polyols, and sugars in cells under salt stress [5]. Among them, proline has been studied most in salt stress. The overexpression of *P5CS1*, which is closely related to the proline synthesis pathway, can significantly improved the salt tolerance of plants [32]. In our study, *BnP5CS* (*BnaA03g18760D*) was induced by salt stress in the two rapeseed varieties and the expression of *BnProDH1* (*BnaAnng07910D*), which encodes proline dehydrogenase, was significantly suppressed under salt stress. The down-regulation of *ProDH* and up-regulation of *P5CS* would make glutamate flow more towards proline, which is in agreement with other studies [33,34]. It is worth noting that more proline was detected in the salt-tolerant variety H62 under salt stress, while no significant difference in proline content in the sensitive variety ZS11 was detected, suggesting that the difference in proline synthesis may lead to the different salt tolerance between H62 and ZS11. In addition, increasing the concentration of sucrose in cells could also regulate osmotic stress [35]. The results of transcriptomics and metabolomics analysis showed that the concentration of sucrose increased significantly under salt stress, and the expression of *Sus3* (sucrose synthase 3) was significantly induced by salt stress in the two rapeseed varieties, suggesting that rapeseed can reduce salt damage by synthesizing sucrose in cells. Fructans were also detected to be synthesized in large amounts under salt-stress conditions, and the increase in salt-tolerant material H62 was more than that of ZS11. Inositol is also a small molecule that regulates osmosis in cells. The metabolome results showed that the content of inositol was significantly increased, and the increase in inositol content in H62 was significantly higher than that of ZS11. Myo-inositol-3-phosphate synthase (*MIPS*) catalyzes the first step of myo-inositol biosynthesis, and its over-expression enhances salt stress tolerance in rice [36]. The transcriptome results showed that *BnaC01g00680D* (*MIPS*) was increased significantly by salt stress.

The balance of intracellular Na^+^/K^+^ concentration is the key to ensuring plants’ normal physiological metabolism under salt stress [37]. At present, several types of ion transporters and ion channels in plants have been confirmed to be involved in the regulation of plant salt stress, such as the NHX family of vacuolar Na^+^/H^+^ antiporters [38], potassium/sodium transporter (HKT-type) [5], and the potassium channel (KAT) [39]. In this study, some DEGs related to ion balance were identified, such as *NHX4* (*BnaC09g19230D*)*, HKT1* (*BnaC02g29240D*), and *KAT1* (*BnaC09g19230D*). Among them, the expression of *HKT1* in two rapeseed varieties under salt stress were both down-regulated greatly, which can limit the absorption of Na^+^ by the cell membrane. Under NaCl stress, the expression of *NHX4* significantly increased, which can force cells to transport more Na^+^ into the vacuole to reduce ion toxicity. The expression of *KAT1* in the two rapeseed varieties was reduced, which may be due to the presence of excessive Na^+^ outside the cell [40]. The expression of *NHX4* of H62 was up-regulated under 150 mM and 200 mM NaCl, which were significantly higher than that of ZS11, indicating that the difference of *NHX4* expression may be one of the reasons for the difference salt tolerance of the two varieties.

Osmomodulin (OSM) is widely present in various tissues of different plants. It is a type of protein synthesized in order to adapt to osmotic pressure. When plant cells are subjected to osmotic stress, the osmomodulin in their cells can absorb water and reduce excessive water loss by changing the membrane’s water permeability. Aquaporins (AQPs) are a class of integral membrane proteins that efficiently and specifically transport water molecules and play an important role in plant water transfer [41], including Plasma membrane intrinsic proteins (PIPs), Tonoplast intrinsic proteins (TIPs), NLM proteins (Nodulin 26-like MIPs, NLMs), and Small and basic intrinsic proteins (SIPs) [42,43,44]. Many studies have shown that aquaporin can be upregulated by salt stress, and the over-expression of related genes can significantly improve the salt tolerance of plants [45,46,47]. In our study, one candidate gene *BnaC03g32130D* (*PIP1-1*) and three candidate genes (*BnaA06g12030D*, *BnaA07g30640D*, *BnaA09g44820D*) homologous to aquaporin *TIP3-1* were identified, all of which can be highly induced by salt stress, and their expression was up-regulated more as the salt concentration increased. The LEA protein (Late-embryogenesis-abundant protein) is widely present in higher plants and accumulates largely during the later stage of seed embryogenesis [48]. Under osmotic stress, the accumulation of a large amount of LEA protein in plants can alleviate cell damage caused by reduced water potential [49,50]. For example, studies have transferred exogenous LEA genes into tobacco, and the salt tolerance and drought resistance of transgenic plants have been identified and the salt tolerance and drought resistance of transgenic tobacco plants have been improved [51]. In our study, *BnaA01g10880D* (*LEA1*), *BnaA09g43150D* (*LEA*), and *BnaC01g35900D* (*LEA76*) were highly induced by salt stress, and their expression was up-regulated more as the salt concentration increased. Dehydrins, part of LEA family, are a group of proteins believed to play a fundamental role in plant response and adaptation to abiotic stresses that lead to cellular dehydration [52]. Therefore, they can alleviate the impact caused by osmotic water loss when exposed to salt stress during the seed-germination stage. In this study, the expression of dehydrohydrin *Rab18* (*BnaC09g08130D*) was highly induced by salt stress, and it was more up-regulated in H62. Non-specific lipid transporter (nsLTP) is an important type of small alkaline-secreted protein in plants, which plays an important role in plant resistance to adversity and stress [53]. In this study, a number of candidate genes homologous to *LTPs* were also identified, and expression levels of *LTPs* were up-regulated significantly as the salt concentration increased in the salt-tolerant H62 (Figure S3). In order to verify the function of the family genes, *BnLTP3* was selected and transferred to *Arabidopsis thaliana*. The results showed that overexpression of *BnLTP3* could increase tolerance to salt stress in *Arabidopsis* plants, but whether the *BnLTP3* has the same function in rapeseed needs further verification. It is worth noting that the root length of over-expressed *Arabidopsis thaliana* under salt stress was significantly higher than that of the wild type under salt stress (Figure 9 and Figure S4), which is consistent with the phenotypes of H62 and ZS11 (Figure 1), indicating that *LTP3* may be involved in promoting root development under salt stress.

Enhancement of antioxidant systems such as catalases (CAT), superoxide dismutase (SOD), ascorbate peroxidase (APX), and glutathione reductase (GR) can increase salt-stress tolerance in plants [7]. In this study, *SOD1* (LOC106346305) was increased in H62_T1, H62_T2, and ZS_T1, and the amount of up-regulation in H62 was significantly higher than that in ZS11. *POD34* (LOC106447805) was increased in H62 and ZS11 under salt stress, and the amount of up-regulation in H62 was significantly higher than that in ZS11. 1-cys peroxiredoxin (*PER1*) is a seed-specific antioxidant that eliminates ROS with cysteine residues [54,55]. *BnaAnng40060D*(*PER1*) was increased in both H62 and ZS11 under salt stress. The results showed that increasing the activity of antioxidant enzymes to resist salt stress is also one of the important mechanisms for rapeseed to resist salt stress, and the expression levels of related genes in salt-tolerant rapeseed were significantly higher than salt-sensitive rapeseed.

Transcription factors (TFs) also play a vital role in response to salt stress in plants, such as *bZIP* [56], *NAC* [57], *WARY* [58], *MYB* [59], and *ERFs* [60]. In this study, several transcription factors induced by salt stress were also identified, such as *BnaC03g31750D* (*MYB-like ETC3*), *BnaA07g21980D* (*ERF018*), and *BnaC03g44820D* (B3 domain-containing transcription factor *ABI3*). These transcription factors can be induced by salt stress and may play a role in the salt tolerance mechanism of rapeseed. Ethylene-responsive factors (ERFs), within a subgroup of the AP2/ERF transcription factor family, are involved in diverse plant reactions to biotic or abiotic stresses [61]. Over-expression of *SlERF1* in tomato plants enhanced salt tolerance during tomato seedling root development, and *SlERF1* activated the expression of stress-related genes, including *LEA*, *P5CS* in tomato plants under salt stress [62]. Overexpression of *BrERF4* from *Brassica rapa* increased tolerance to salt and drought in *Arabidopsis* plants [60]. In this study, *BnaC05g00680D* (*ERF023*) and *BnaA07g21980D* (*ERF018*) were highly induced by salt stress, and were more up-regulated in H62 under salt stress, implying that these gene have a certain relationship with the difference in salt tolerance between the two rapeseed varieties.

In addition, there are some other genes and metabolites also involved in the regulation of salt tolerance. For example, NADP-malic enzyme functions in many different pathways in plant and may be involved in plant defense, such as salt stress [63]. Transgenic *Arabidopsis* plants over-expressing rice *NADP-ME1* had a greater salt tolerance at the seedling stage than wild-type plants [64]. In our study, *NADP-ME1* (*BnaA07g00860D*) was significantly up-regulated in two rapeseed varieties under salt stress, and the amount of up-regulation in H62 was significantly higher than that in ZS11. Flavonoids are a large group of secondary plant metabolites, playing diverse roles in plant growth, development, and responses to stress. Epigallocatechin-3-gallate (EGCG, a bioactive flavonoid) was found to enhance tolerance to salt stress [65], and EGCG could alleviate salt stress-induced inhibition in seed germination and root growth in tomato [66]. In our study, the content of EGCG was significantly increased in both H62 and ZS11, and the increase in EGCG content in H62 was significantly higher than that of ZS11.

**Figure 10:**
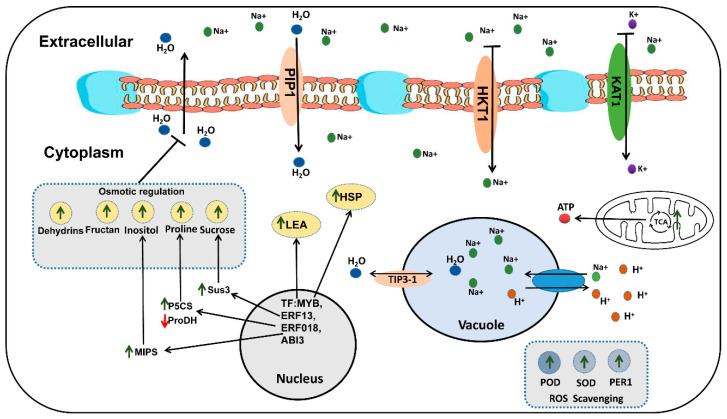
Possible molecular mechanism of salt tolerance in rapeseed.

Based on the data results of the correlation analysis of key DEMs and DEGs (Figure S5), some putative mechanisms of rapeseed salt tolerance were (Figure 10): (1) High concentrations of NaCl in the environment decreased the water potential, which made it difficult for the plant cells to absorb external water. In order to reduce the harm of osmotic stress, the genes related to osmotic regulation in rapeseed is regulated by related transcription factors (MYB, ERF, bHLH) in the nucleus. Salt stress can promote the TCA cycle of rapeseed and produce more ATP for rapeseed metabolic activity. The up-regulation of *P5CS* and down-regulation of *ProDH* increased proline synthesis, the up-regulation of *Sus3* and *MIPs* increased Sucrose and Inositol synthesis, and macromolecular proteins, such as LEA, Dehydrins, began to accumulate in large quantities, which could balance the water potential inside and outside the cells to some extent. In addition, the PIP protein located on the cell membrane and the TIP protein located on the vacuole membrane can also regulate the water balance in the cell and the vacuole. (2) High concentrations of Na^+^ inhibit the activity of *KAT1*, thereby inhibiting the absorption of K^+^. A large amount of Na^+^ enters the cells, and then causes ion toxicity in the cells. The down-regulation of *HKT1* can limit the absorption of Na^+^ by the cell membrane, and the up-regulation of NHXs can force cells to transport more Na^+^ into the vacuole to reduce ion toxicity. (3) Large amounts of ROS were generated in cells under salt stress, which may cause oxidative damage to cells. The ROS scavenging system may neutralize the excess ROS. The up-regulation of the ROS scavenging system-related genes (such as *POD*, *SOD*, and *PER1*) decreased ROS content in the cell to reduce its damage to the cell. In addition, the enhanced TCA cycle provides energy for gene transcription, translation, and substance synthesis under salt stress. The functional genes mentioned above need further functional verification.

## 4. Materials and Methods

### 4.1. Plant Material, Salt Stress Treatment, and Sample Collection

The experimental materials included Huayouza 62 (H62) and Zhongshuang 11 (ZS11). The seed germination experiment method was performed as previously described [67]. Thirty healthy seeds of H62 and ZS11, full and of the same size, were selected, surface-sterilized with 0.1% HgCl solution for 3 min, rinsed with distilled water three times, and equally distributed into Petri dishes (9 cm in diameter) containing two sheets of medical gauze. 15 mL of the NaCl solutions (0, 100, 150, and 200 mM) were added to the respective Petri dishes. The seed germination experiment was performed in a greenhouse (20 °C), with an 8 h day, 16 h night photoperiod.

On the 8th day after sowing, 8–10 seedlings with relatively consistent growth were selected for each treatment, and the hypocotyl length and main root length were measured; 3 seedlings were selected for each treatment and mixed into a biological sample, which were stored in the refrigerator at −80 °C by liquid nitrogen quick freezing. Finally, Huayouza 62 took three groups of samples, named H62_CK, H62_T1, and H62_T2, and Zhongshuang 11 took two groups of samples, named ZS_CK and ZS_T1, in which CK was 0 mM, T1 was 150 mM, and T2 was 200 mM. The samples were determined and analyzed by BGI. Finally, there were 3 and 7 biological repeats for the transcriptomics and metabolomics analysis, respectively.

### 4.2. LC-MS for Metabolite Determination

The tissue samples were stored at −80 °C for metabolomics analysis. We weighed 25 mg tissue samples into Eppendorf Micro Test Tubes (EP tubes), added 800 μL of pre-cooled (4 °C) methanol/water (1:1) buffer solution to each EP tube, added two tiny steel balls to each EP tube, and placed the sample in TissueLyser Medium grinding (QIAGEN, Düsseldorf, Germany), setting the parameter to 50 HZ, 4 min; then, we removed the steel balls after grinding, placed the centrifuge tube in a refrigerator at −20 °C for overnight precipitation, and centrifuged at 30,000 *g* for 20 min (4 °C). We then carefully removed the EP tube from the centrifuge, and drew 550 μL of each sample; GC-MS analysis was performed in a new EP tube.

### 4.3. LC-MS (Gas Chromatography-Mass Spectrometry) Analysis

The LC-MS acquired all samples, and the system followed machine orders. Firstly, all chromatographic separations were performed using an ultra-performance liquid chromatography (UPLC) system (Waters, UK). An ACQUITY UPLC HSS T3 column (100 mm * 2.1 mm, 1.8 μm, Waters, UK) was used to reverse phase separation. The column oven was maintained at 50 °C. The flow rate was 0.4 mL/min and the mobile phase consisted of solvent A (water + 0.1% formic acid) and solvent B (acetonitrile + 0.1% formic acid). Gradient elution conditions were set as follows: 0~2 min, 100% phase A; 2~11 min, 0% to 100% B; 11~13 min, 100% B; 13~15 min, 0% to 100% A. The injection volume for each sample was 10 μL. A high-resolution tandem mass spectrometer Xevo G2 XS QTOF (Waters, UK) was used to detect metabolites eluted from the column. The Q-TOF was operated in both positive and negative ion modes. The mass spectrometry data were acquired in Centroid MSE mode. The TOF mass range was from 50 to 1200 Da and the scan time was 0.2 s. For the MS/MS detection, all precursors were fragmented using 20–40 eV, and the scan time was 0.2 s. During the acquisition, the LE signal was acquired every 3 s to calibrate the mass accuracy. Furthermore, to evaluate the LC-MS stability during the whole acquisition, a quality control sample (pool of all samples) was acquired after every 10 samples. Original data were preprocessed, peak list information was extracted, and data correction was performed. Peak extraction was mainly achieved through the commercial software Progenesis QI (version 2.2), which includes peak alignment, peak extraction, normalization, deconvolution, and compound identification. Local polynomial regression fitting signal correction (QC-RSC) based on QC sample information for real sample signals is a more effective data correction method for omics analysis in the field of metabolomics [68].

### 4.4. Metabolite Data Analysis to Obtain Differential Metabolites

The main purpose of metabolomics analysis was to screen statistically and biologically significant metabolites from the mass metabolites detected and clarify the metabolic process and the changing mechanism of the organism based on this. Single and multi-dimensional methods were used to analyze from different angles according to the characteristics of the data. Univariate analysis was performed using *t*-test and fold change analysis (FC analysis). Further FDR correction was performed in the statistical analysis process on the *p*-value produced by the statistical test to obtain the q-value. Multivariate analysis was performed using principal component analysis (PCA) and partial optimal multiplication-discrimination analysis (PLS-DA). PCA was mainly used to observe the trend of separation between groups in the experimental model, specifically, whether any abnormal points appeared and reflected the variability between and within groups from the original data. PLS-DA uses partial maximum regression to establish a relationship model between metabolite expression and sample categories to achieve model prediction of sample categories. At the same time, the variable projection important degree (Variable Important for the Projection, VIP) was used to measure the influence intensity and explanatory power of each metabolite expression pattern on the classification and discrimination of each group of samples, thereby assisting the selection of metabolic markers. In this study, the ion identification data in positive mode were selected. Conditions for screening differentially expressed metabolites (DEMs): (1) VIP ≥ 1; (2) Fold-change ≥ 1.2 or Fold-change ≤ 0.833; (3) q-value < 0.05. The three were taken to intersect, and the obtained substance was regarded as a substance with a significant change in content. Determination of metabolites and analysis of metabolic pathways were based on the KEGG database.

### 4.5. Transcriptome Sequencing Analysis

Fifteen libraries representing the plant from the two lines collected at three NaCl concentrations and in three replicates were constructed for transcriptome sequencing. Total RNA was extracted using a TIANGEN RNA Prep Pure Plant kit (Tiangen Biotech Co., Ltd., Beijing, China) and purified with the DNase I, RNase-free, and Thermo Scientific (Waltham, MA, USA) RebertAid First Strand cDNA Synthesis Kit. RNA concentration was measured using Agilent 2100 Bioanalyzer (Agilent Technologies, Santa Clara, CA, USA) and NanoDropTM (Thermo Scientific, Waltham, MA, USA) evaluated the purity of RNA. Sequencing was performed using an Illumina HiSeqTM paired-end sequencing system (San Diego, California, USA). It contains crucial steps such as mRNA purification, mRNA fragmentation, adaptor addition, reverse transcription, and library validation.

### 4.6. Sequencing Analysis

The sequencing reads, which contain low-quality, adaptor-polluted and high content of unknown base (N) reads, should be processed to be removed before downstream analysis. After reads filtering, the clean reads were mapped to the reference genome (ZS11: https://www.ncbi.nlm.nih.gov/assembly/GCF_000686985.2/, accessed on 10 April 2019) using HISAT2 (v2.0.4) software [69]. Fragments per kilobase per million mapped fragments (FPKM) were calculated to estimate the expression level of genes in each sample [70]. DESeq2 was used to estimate the expression level of each gene in each sample [71]. In this experiment, *p*-value ≤ 0.05 and |log_2_FoldChange| ≥ 1 were set as the thresholds to determine the significance of the gene-expression difference between samples. Notably, gene-expression comparisons of the samples at the 0, 150 mM, and 200 mM concentrations (H62_T1/H62_CK, H62_T2/H62_CK, and ZS11_T1/ZS11_CK) were performed. Gene Ontology (GO) annotation (http://geneontology.org/, accessed on 28 May 2019) and Kyoto Encyclopedia of Genes and Genomes (KEGG) reference pathway (https://www.genome.jp/kegg/kegg.html, accessed on 4 June 2019) analyses were performed for DEGs.

### 4.7. RT-PCR and Real-Time Quantitative PCR

In order to validate the reliability of our RNA-seq data, we measured mRNA abundance using qRT-PCR for 6 DEGs (Table S1). Three biological replications of RNA from the samples (H62 and ZS11) at the 0, 150 mM, and 200 mM concentrations were used for qPCR analysis. The gene-specific primers for real-time PCR analysis were designed using Primer 3 by applying the parameters described by Thornton and Basu [72]. The first-strand cDNAs were synthesized from 1 μg of total RNAs using SuperScript III Reverse Transcriptase (Invitrogen, Carlsbad, CA, USA). Ten microliters of PCR samples containing 1 μL of first-strand cDNAs and 5 pmol of primers were then subjected to 30 cycles of 30 s denaturing at 94 °C, 30 s annealing at 60 °C, and 30 s extending at 72 °C. The PCR products were electrophoresed on 1.5% agarose gel. Real-time PCR was performed on CFX Connect Real-Time PCR Detection System (Bio-Rad, Hercules, CA, USA) using 1 μL of cDNAs and SsoAdvanced SYBR Green Supermix (Bio-Rad). The thermal conditions were set at 95 °C for 3 min denaturation, followed by 40 cycles of 95 °C for 1 s and 60 °C for 30 s. Following denaturation at 95 °C for 30 s and cooling to 65 °C for 30 s, a melting curve was generated by heating from 65 °C to 95 °C in 0.5 °C increments with a dwell time at each temperature of 2 s while continuously monitoring the fluorescence. All of the reactions were performed in triplicate and the average expression value was calculated. The relative expression level for each gene was calculated using the 2^−ΔΔC_T_^ method with normalization to the internal control gene [73].

### 4.8. BnLTP3 Gene Function Verification

The Col-0 ecotype of *Arabidopsis thaliana* was used as the WT (wild-type). Plants were grown at 23 °C with a long-day light cycle (16 h light/8 h dark). *BnLTP3* (*BnaC03g12050D*) cDNA sequences were cloned, and the overexpression vector pC2300 were driven using a CaMV 35S promoter. The vectors were introduced into the *Agrobacterium tumefaciensstrain* GV3101 for genetic transformation into Col-0 ecotype of *Arabidopsis thaliana*. Homozygous mutants were confirmed by PCR-based genotyping. The T2 generations *Arabidopsis* seeds were certified, and uniform and healthy seeds were surface-sterilized with sodium hypochlorite solution and then rinsed in sterile distilled water. The seeds were germinated on 1/2 MS and 1/2 MS + NaCl (50, 100 mM) basal medium containing 2% (*w*/*v*) sucrose. After 10 days, 3 relatively uniform seedlings were selected to measure root length and RNA was extracted to detect the expression of *LTP3* gene (F:GTTCCTCCTCCGTGTTGTG, R:TTGTCGCAGTTAGTGCTCAC).

## 5. Conclusions

A total of 103 key differentially expressed metabolites (DEMs) involved in TCA cycle, proline metabolism, inositol metabolism, carbohydrate metabolic processes, and oxidation-reduction processes and 53 key differentials expressed genes (DEGs) involved in ion transport, reactive oxygen scavenging, osmotic regulation substance synthesis, and macromolecular protein synthesis were identified. The overexpression of *BnLTP3* could increase tolerance to salt stress in *Arabidopsis* plants.

## Figures and Tables

**Table 1:** Summary of the sequencing results.

Sample	TRR	TCR	CRR	TMR	UMR	TGN	KGN
H62_CK_1	54.04	45.39	84.01	73.44	21.63	77,996	72,412
H62_CK_2	54.04	44.55	82.44	71.43	21.90	79,639	73,968
H62_CK_3	54.03	44.55	82.44	71.71	21.35	77,173	71,656
H62_T1_1	54.03	44.18	81.76	71.6	21.01	77,378	71,886
H62_T1_2	54.04	44.35	82.08	73.06	20.64	77,245	71,684
H62_T1_3	55.67	44.71	80.30	74.1	20.98	75,820	70,371
H62_T2_1	54.03	44.84	82.98	76.88	19.52	72,893	67,757
H62_T2_2	54.03	44.76	82.84	75.4	21.7	75,294	69,933
H62_T2_3	54.03	44.22	81.83	75.19	20.19	75,088	69,839
ZS11_CK_1	54.03	44.56	82.46	74.48	21.96	76,629	71,160
ZS11_CK_2	54.04	45.22	83.69	74.13	22.2	77,841	72,342
ZS11_CK_3	54.03	44.28	81.94	73.32	21.53	76,138	70,811
ZS11_T1_1	54.03	44.13	81.68	75.23	22.35	75,167	69,754
ZS11_T1_2	57.14	45.15	79.00	76.26	22.34	72,940	67,636
ZS11_T1_3	57.14	45.30	79.28	75.21	22.64	73,875	68,597

TRR: Total Raw Reads (Mb); TCR: Total Clean Reads (Mb); CRR: Clean Reads Ratio (%); TMR: Total Mapping Ratio; UMR: Uniquely Mapping Ratio; TGN: Total Gene Number; KGN: Known Gene Number; Seedlings of two genotypes with differential salt tolerance, H62 (tolerant) and ZS11 (sensitive), were sampled for RNA sequencing after exposure to 0 (CK), 150 mM (T1), 200 mM NaCl (T2) for 8 days.

## Data Availability

Not applicable.

## Supplementary Materials

The following supporting information can be downloaded at: https://www.mdpi.com/article/10.3390/ijms23031279/s1.
