# Supplementary material for: Combined Transcriptomics and Metabolomics Analysis Reveals the Molecular Mechanism of Salt Tolerance of Huayouza 62, an Elite Cultivar in Rapeseed (Brassica napus L.)
Source: Int J Mol Sci. 2022 Jan 24;23(3):1279. doi: 10.3390/ijms23031279 (PMC8836002; doi:10.3390/ijms23031279)
Supplement: Supplementary file 1 [file ijms-23-01279-s001.zip › ijms-1559644-supplementary.pdf]

Supplementary data

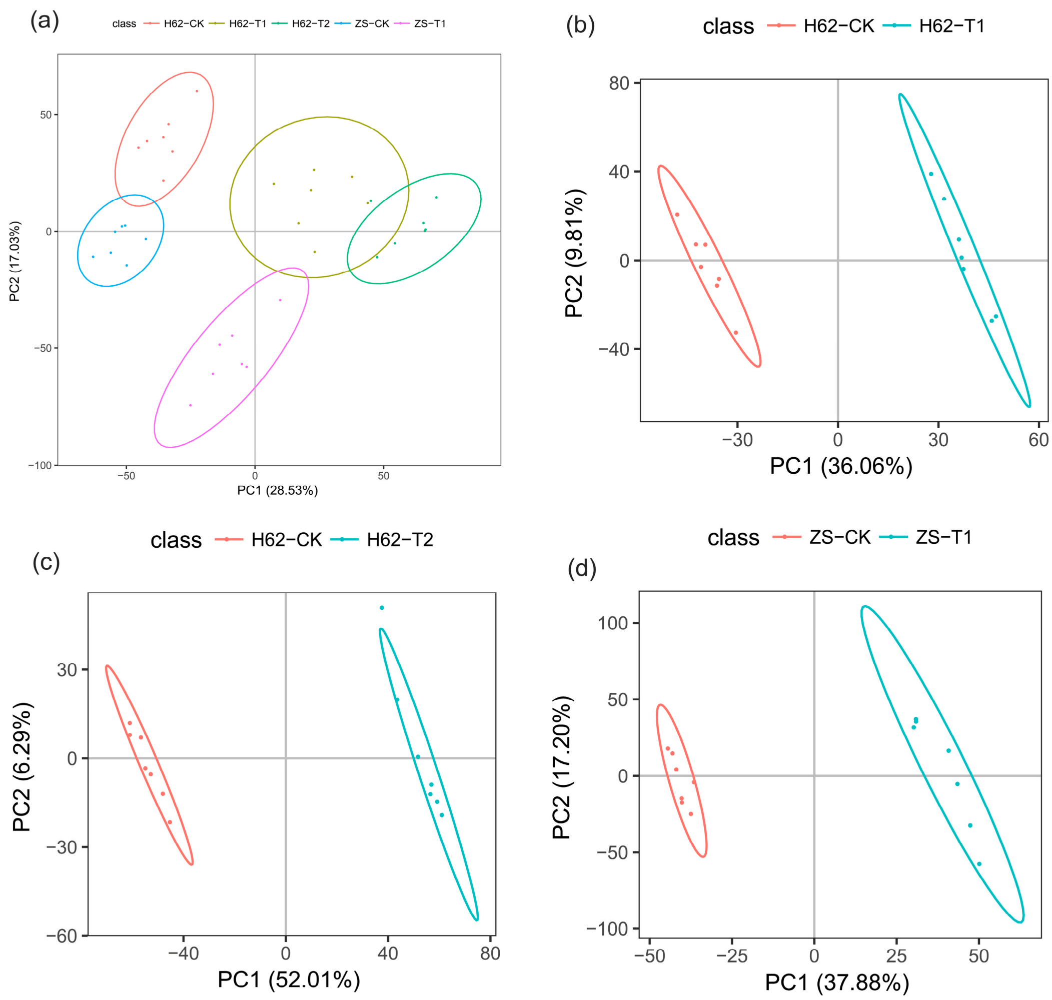

Figure S1. PCA and PLS-DA scatter plot of two rapeseed varieties under different treatments.

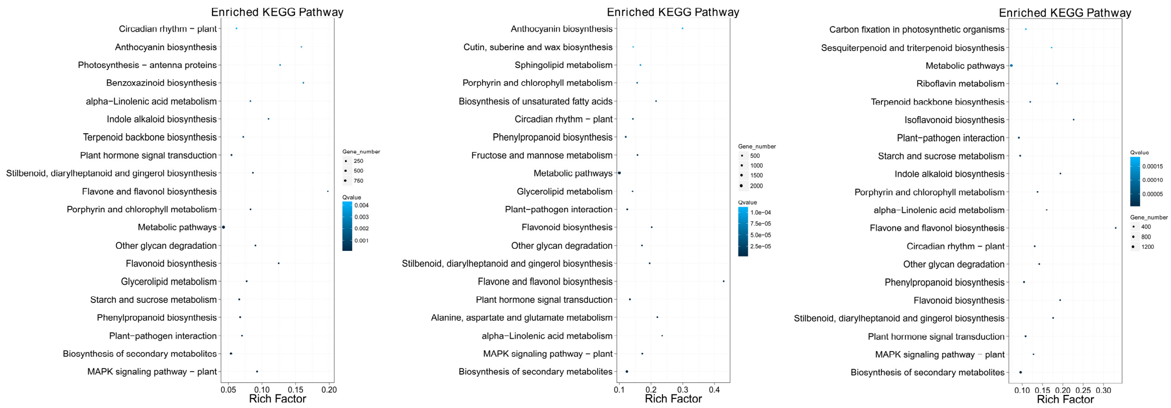

H62_T1 VS H62_CK H62_T2 VS H62_CK ZS11_T1 VS ZS11_CK

Figure S2. The KEGG pathways the DEGs in H62_T1 VS H62_CK, H62_T2 VS H62_CK, ZS11_T1 VS ZS11_CK.

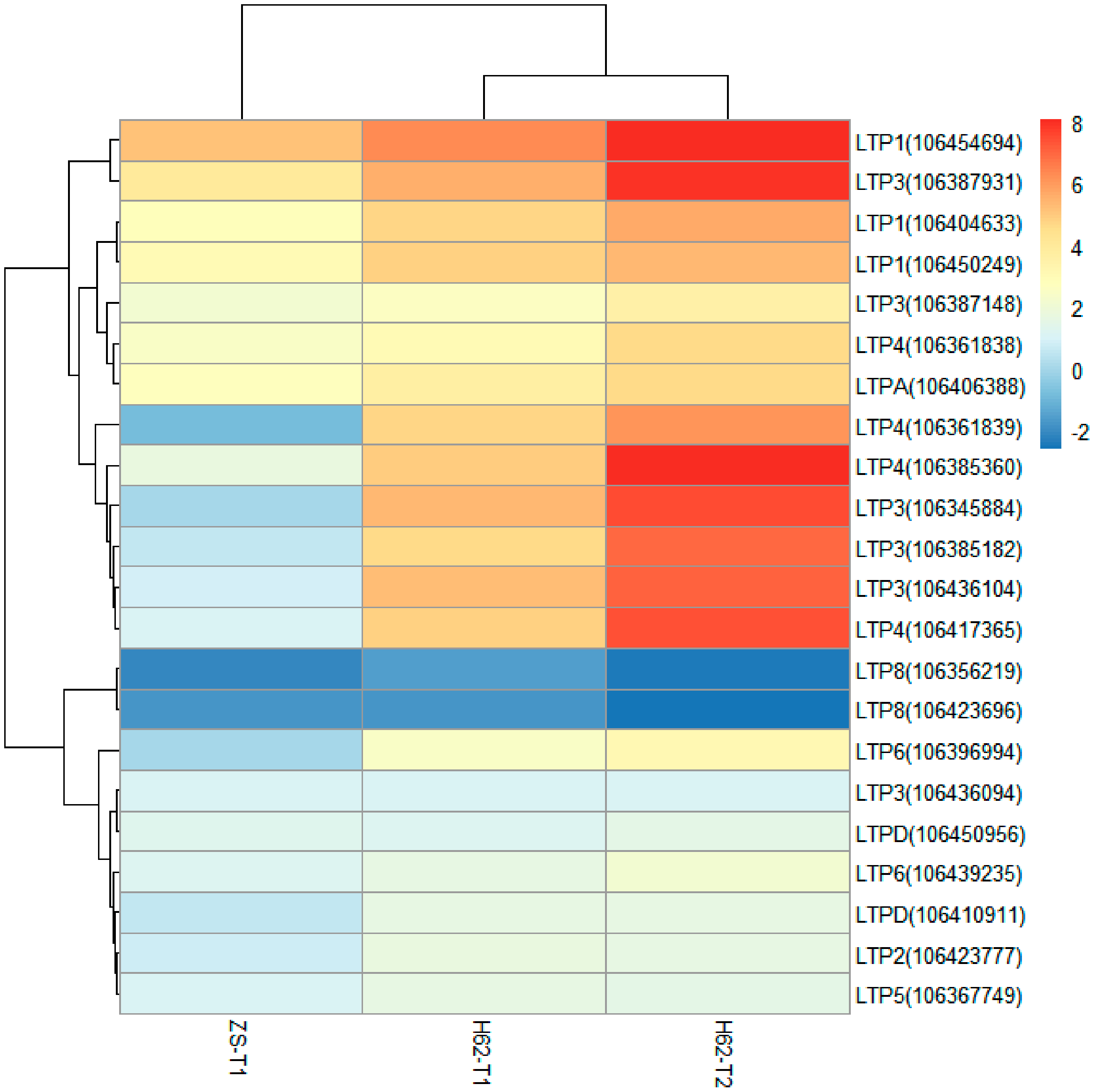

Figure S3. The heat map of nsLTP genes expression pattern two rapeseed varieties under different treatments base on the log_2Foldchange.

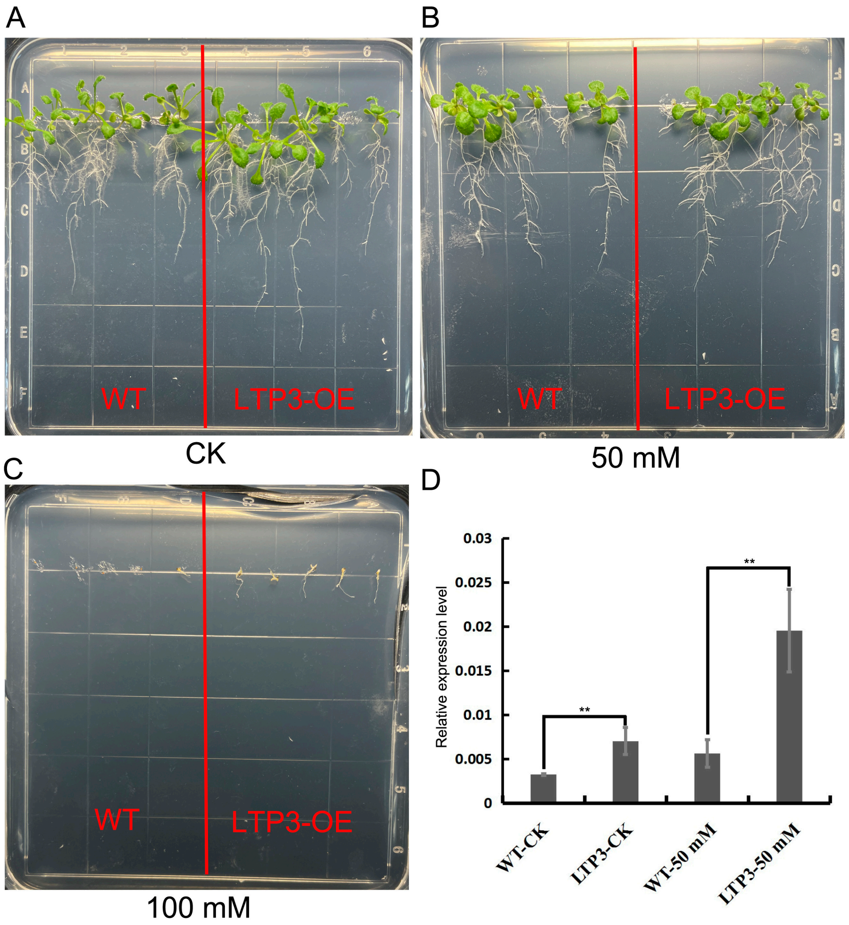

Figure S4. Over-expression of *BnLTP3* in *Arabidopsis thaliana*.

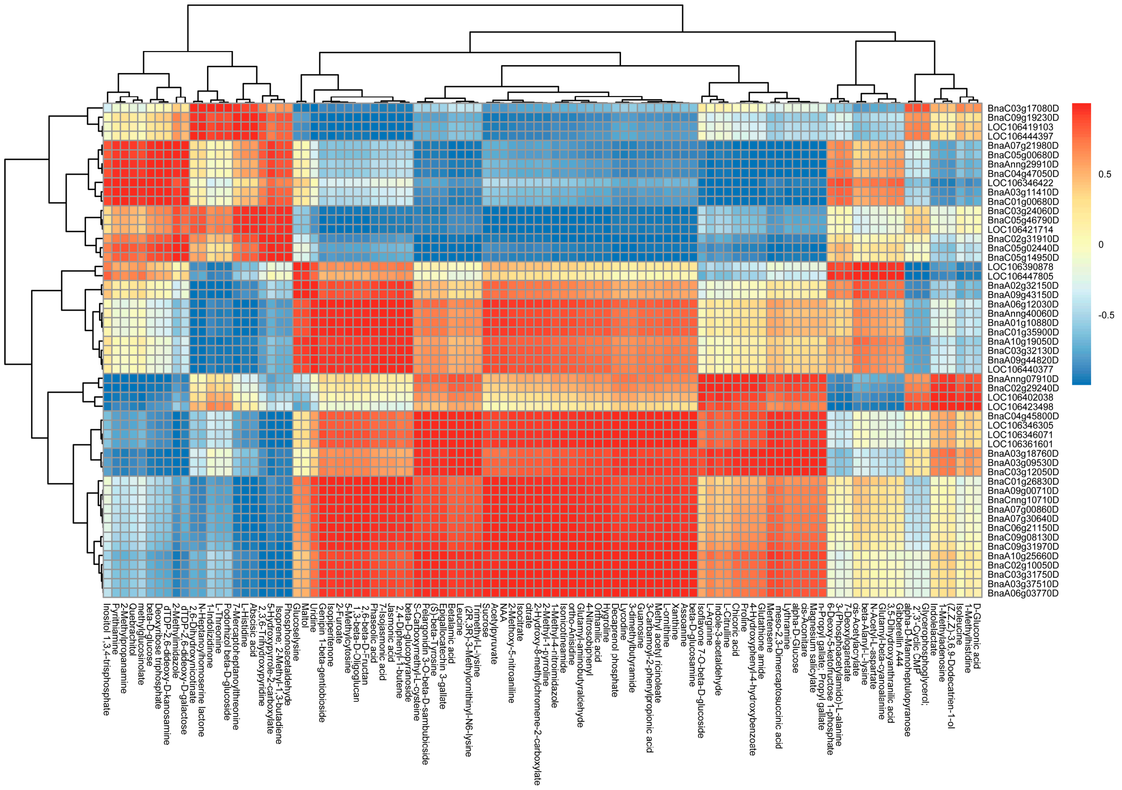

Figure S5. The correlation analysis of key DEMs and DEGs.

Table S1. The primer sequence of qPCR verification gene.

Gene ID	***B.napus*** **name**	Forward Primer	Reversed Primer	PCR product Lenth (bp)
106390833	BnaA01g27710D	GCACGATGACAGCACTA	CTCAGCACTTGACCTCC	100
106415866	BnaC07g17990D	CTGAGGCTCGTACTTACC	AACCACCCAAATCTTCC	233
106347720	BnaC05g27530D	AATGCTACTTTCAATCGCTAC	GCTGTTCCTCCTCTGATACTC	264
106381333	BnaA02g12330D	TCTTCAGACAGCGTTTCATT	GGAGATAGATCCGTCAGGTAC	126
106401061	BnaC05g15180D	ATCAACATCTCCGACAAACC	CCTTCAGGACTAATGGCTAA	50
106436104	BnaA03g09530D	GTTCCTCCTCCGTGTTGTG	TGTCGCAGTTCGTGCTCAT	199

Table S2. Kyoto Encyclopedia of Genes and Genomes (KEGG) pathway analysis of the DEMs.

Table S3. The key DEMs responding to salt stress.

Table S4. Kyoto Encyclopedia of Genes and Genomes (KEGG) pathway analysis of the DEGs.

Table S5. The candidate genes related to salt stress.
